# Single immunization with an influenza hemagglutinin nanoparticle‐based vaccine elicits durable protective immunity

**DOI:** 10.1002/btm2.10689

**Published:** 2024-06-03

**Authors:** Shiho Chiba, Tadashi Maemura, Kathryn Loeffler, Steven J. Frey, Chunyang Gu, Asim Biswas, Masato Hatta, Yoshihiro Kawaoka, Ravi S. Kane

**Affiliations:** ^1^ Department of Pathobiological Sciences, School of Veterinary Medicine Influenza Research Institute, University of Wisconsin‐Madison Madison Wisconsin USA; ^2^ Pandemic Preparedness, Infection and Advanced Research Center (UTOPIA) The University of Tokyo Tokyo Japan; ^3^ School of Chemical and Biomolecular Engineering, Georgia Institute of Technology Atlanta Georgia USA; ^4^ Division of Virology, Department of Microbiology and Immunology Institute of Medical Science, University of Tokyo Tokyo Japan; ^5^ The Research Center for Global Viral Diseases, National Center for Global Health and Medicine Research Institute Tokyo Japan

**Keywords:** adjuvant, duration of immunity, ferret, influenza A virus, neutralizing antibody titers, virus‐like particles (VLPs), VLP‐based vaccine

## Abstract

Vaccination is the most effective strategy to combat influenza. Ideally, potent and persistent vaccine effects would be induced with a single vaccine dose. Here, we designed a virus‐like particle (VLP)‐based vaccine presenting multiple copies of the influenza hemagglutinin (HA) from A/Puerto Rico/8/1934 (PR8HA‐VLP) and examined its immunogenicity and protective efficacy in ferrets. Serum‐neutralizing antibodies were effectively induced against the homologous virus at 3‐week post‐vaccination with a single dose of PR8HA‐VLP with or without adjuvants. When the single‐immunized ferrets were challenged with the homologous virus, virus replication in the nasal mucosa was significantly reduced. Long‐term monitoring of serum titers revealed that after adjuvanted vaccination with PR8HA‐VLP, neutralizing antibodies were retained at similar levels 20‐ to 183‐week post‐vaccination, although a 4‐ to 8‐fold titer decline was observed from 3‐ to 20‐week post‐vaccination. Boost immunization at 183 weeks after the first immunization elicited higher neutralizing antibody titers than those at 3 weeks after the initial immunization in most of the animals. These results confirm that nanoparticle‐based vaccines are a promising approach to effectively elicit durable multiyear neutralizing antibody responses against influenza viruses.

**Translational Impact Statement**

A vaccine that elicits a robust and long‐lasting immune response would be the most effective strategy to combat influenza. We designed a virus‐like particle (VLP)‐based vaccine presenting multiple copies of the influenza protein, HA. Ferrets immunized with a single dose of the vaccine were protected from a viral challenge. High neutralizing antibody titers were retained for three and a half years post‐vaccination. VLP‐based vaccines are thus a promising approach to elicit durable neutralizing antibody responses against influenza viruses.

## 1 INTRODUCTION

Seasonal influenza viruses are respiratory viruses that annually cause morbidity and mortality worldwide. Vaccination is the most potent solution to reduce disease severity upon influenza virus infection. Current influenza vaccines mainly elicit antibodies that target the viral glycoprotein hemagglutinin (HA) to neutralize the viruses. The ideal influenza vaccine would induce potent and persistent neutralizing antibody titers with a single vaccination.

Presenting multiple copies of antigens from virus‐like particle (VLP)‐based scaffolds has emerged as a promising strategy to obtain high levels of neutralizing antibodies, because these scaffolds mimic the size and geometry of natural virus particles.^1^, ^2^, ^3^ VLP‐based vaccines have been approved for clinical use to prevent human infections caused by viruses that include hepatitis B virus (HBV) and human papillomaviruses.^4^ HBV VLP‐based vaccines, Recombivax HB and Engerix‐B, were licensed in the United States in 1986 and 1989, respectively. VLP‐based vaccines have also been approved for protection against human papillomaviruses, including the 9‐valent vaccine Gardasil‐9.^5^, ^6^ Given this demonstrated clinical efficacy of VLP‐based vaccines against viral diseases, we generated a VLP‐based vaccine presenting HA from A/Puerto Rico/8/1934 (PR8HA‐VLP), and examined its immunogenicity and protective effects in ferrets, the gold standard animal model for influenza virus infection.^7^, ^8^

## 2 RESULTS AND DISCUSSION

We previously developed protein nanoparticles composed of the MS2 bacteriophage coat protein^9^ coated with streptavidin (SA) as a method of displaying diverse antigens, including HA as well as the SARS‐CoV‐2 Spike (S) protein and its S2 domain.^10^, ^11^, ^12^, ^13^ MS2 dimers with an AviTag inserted into a surface loop^14^ and SA were expressed in BL21(DE3) *Escherichia coli* (*E. coli*) and purified as previously described.^10^, ^11^, ^12^, ^13^ After purification, MS2 was biotinylated and then added slowly to a large excess of SA to generate MS2‐SA particles.^10^, ^11^, ^12^, ^13^ Excess SA was removed by size exclusion chromatography (SEC).

**FIGURE 1:**
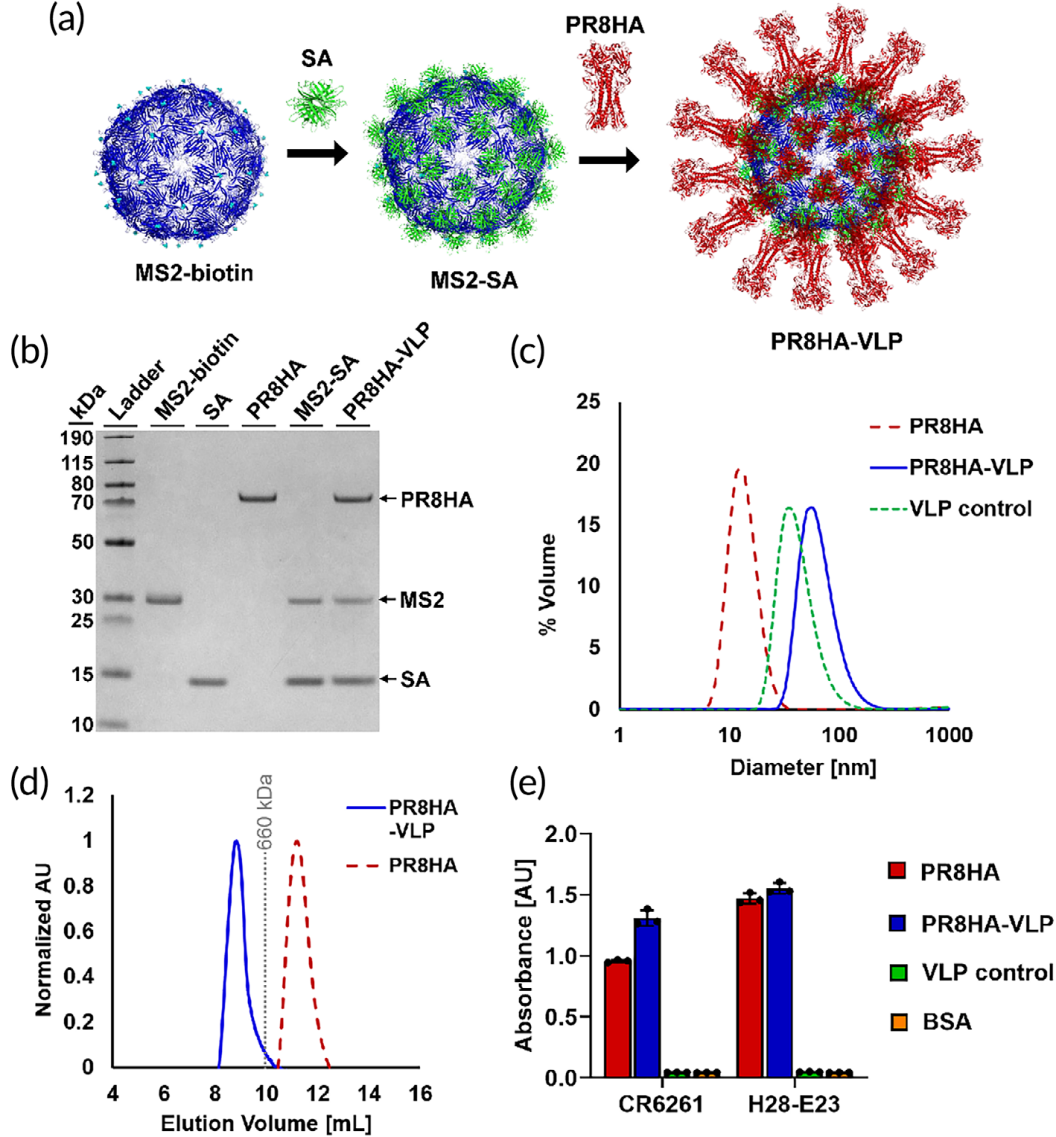
Preparation of PR8HA‐VLP vaccine. (a) Schematic representation of PR8HA‐VLP (MS2‐biotin = dark blue, PDB: 2MS2; SA = green, PDB: 6J6J; PR8HA = red; PDB: 1RU7). (b) SDS‐PAGE characterization of VLP and PR8HA proteins. The unprocessed gel is shown in Supporting Information S1: Figure 2. (c) Characterization of PR8HA (red), VLP control (green), and PR8HA‐VLP (blue) by dynamic light scattering. Curves are an average of five measurements. (d) Size exclusion chromatography traces for PR8HA‐VLP (blue) and PR8HA (red). The dotted gray line represents the elution volume of the molecular weight standard thyroglobulin (660 kDa). The column void volume is 7.2 mL. (e) Characterization of the binding of stalk‐binding antibody CR6261 and head‐binding antibody H28‐E23 (Sb epitope) to PR8HA, PR8HA‐VLP, VLP control, and bovine serum albumin (BSA) (mean ± SD, *n* = 3: one independent assay with three technical replicates). SA, streptavidin; VLP, virus‐like particle.

To display HA on the MS2‐SA VLP, we inserted an AviTag at the C‐terminus of the PR8HA ectodomain (A/Puerto Rico/8/1934 H1N1).^10^ PR8HA was co‐expressed along with the enzyme BirA in High Five insect cells using the baculovirus expression system and purified by immobilized metal affinity chromatography and SEC.^10^ The appropriate ratio of PR8HA to MS2‐SA VLP was determined by mixing various ratios of the proteins and running the mixture on SEC to measure the amount of excess PR8HA.^10^ The ratio with the highest amount of PR8HA on the VLP without any excess soluble HA was selected.^10^ VLP and PR8HA were incubated together for 1 h at room temperature to produce the final vaccine samples (Figure 1a).

The purity and proper structure of each protein were characterized during the various assembly steps. First, MS2, SA, and HA proteins and assembled MS2‐SA and PR8HA‐VLP particles were characterized by SDS‐PAGE to verify protein size and purity (Figure 1b). HA appeared at ~63 kDa, MS2 at ~29 kDa, and monomeric SA at ~15 kDa, matching their expected molecular weights. The final PR8HA‐VLPs were next characterized by dynamic light scattering (DLS). DLS measurements indicate an average diameter of ~43 nm for MS2‐SA VLPs and ~67 nm for PR8HA‐VLP (Figure 1c). To further show the attachment of PR8HA to the VLP, analytical SEC chromatograms were generated for PR8HA‐VLP and for the PR8HA trimer (Figure 1d). The PR8HA‐VLP peak is shifted to the left compared to the PR8HA peak, indicating an increase in size and showing that no excess PR8HA is remaining. Finally, we confirmed the binding of anti‐HA monoclonal antibodies CR6261 and H28‐E23 to PR8HA‐VLP and PR8HA by enzyme‐linked immunosorbent assay (ELISA) (Figure 1e).

**FIGURE 2:**
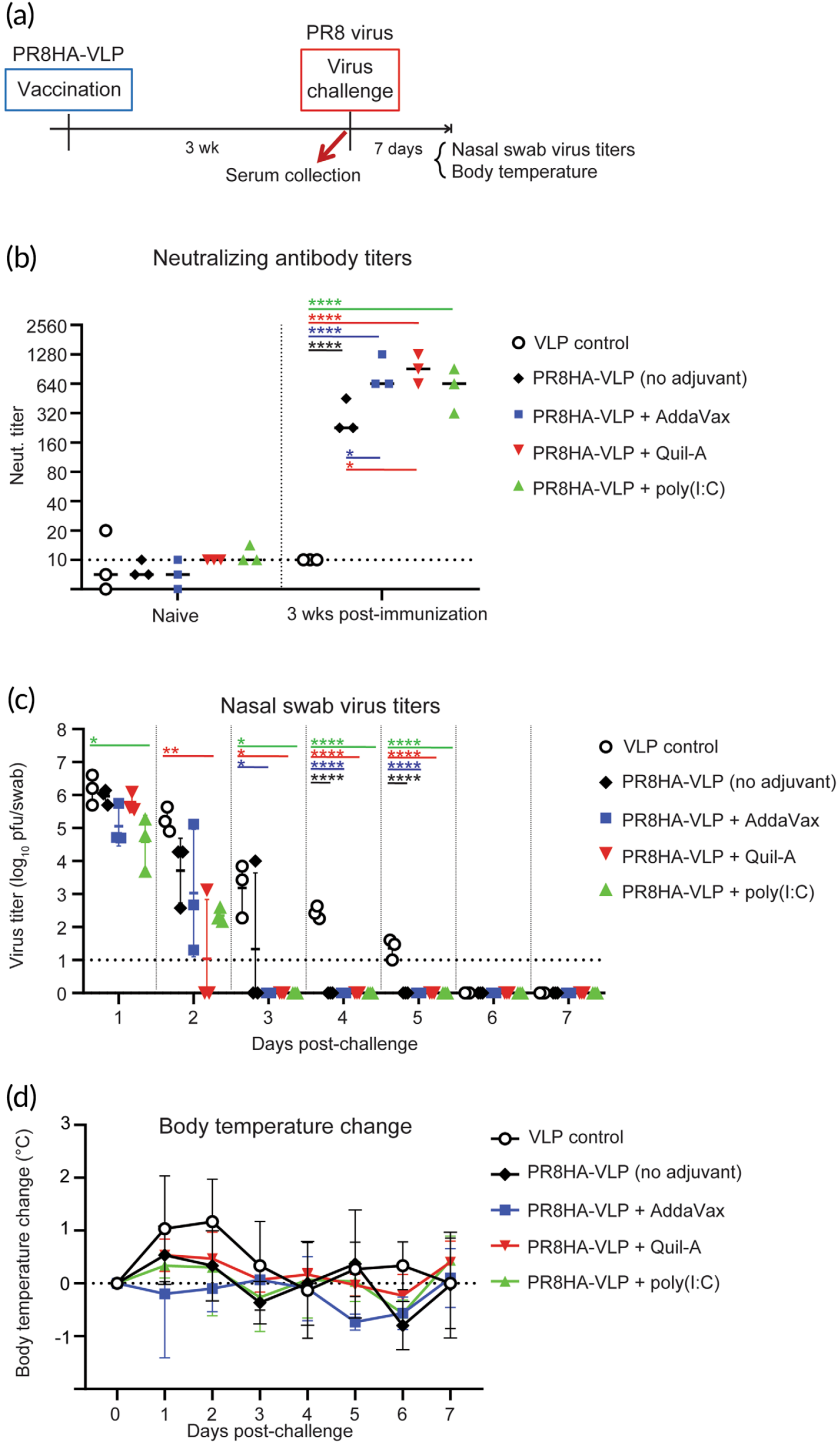
Immunogenicity of PR8HA‐VLP in ferrets. (a) Timeline of the immunization/virus challenge study. Groups of ferrets (*N* = 3/group) were subcutaneously inoculated with MS2‐SA nanoparticles presenting PR8HA (PR8HA‐VLP; HA content: 45 μg) adjuvanted with or without AddaVax (500 μL), Quil‐A (30 μg), or poly(I:C) (3 μg), or inoculated with the same amount of VLP‐only as a control. (b) Serum‐neutralizing antibody titers at 3‐week post‐immunization were analyzed against PR8 virus in micro‐neutralization assays. Data shown are the geometric means of duplicates. Dashed lines represent the detection limit of the assay. Bars show the median of the groups. (c, d) At 3‐week post‐immunization, the animals were intranasally (i.n.) challenged with 10^6^ pfu of homologous PR8 virus. Virus titers in nasal swabs (c) and body temperature increase as a clinical symptom (d) were monitored daily for 7 days post‐challenge. (c) Bars show the median of the groups. (d) Data represent the means and SD of each group (*N* = 3). Statistical analyses were performed by using a one‐way analysis of variance and corrected for multi‐group comparison by using Dunnett's test. SA, streptavidin; VLP, virus‐like particle. *p < 0.05; **p < 0.01; ***p < 0.001; ****p < 0.0001.

We next examined the immunogenicity of PR8HA‐VLP in ferrets (Figure 2a). Ferrets were subcutaneously inoculated with PR8HA‐VLP adjuvanted with or without AddaVax (an MF59‐like squalene‐based oil‐in‐water nano‐emulsion),^15^ Quil‐A (a mixed fraction of plant‐derived saponins), or poly(I:C) (a synthetic analog of double‐stranded RNA and toll‐like receptor 3 and RIG‐I/MDA5 agonist),^16^ which are well‐established adjuvants in pre‐clinical studies and/or equivalents of the ones formulated in commercially available vaccines.^17^, ^18^, ^19^, ^20^ Negative control animals were inoculated with MS2‐SA VLP alone (VLP control). At 3‐week post‐immunization, the animals were bled and neutralizing antibody titers against the homologous PR8 virus were determined by using a micro‐neutralization assay (Figure 2b). A single immunization with PR8HA‐VLP elicited a geometric mean titer (GMT) of 226, even in the absence of any adjuvant (Figure 2b, right). The neutralizing titers were significantly enhanced when PR8HA‐VLP was adjuvanted with AddaVax or Quil‐A with average GMTs of 853 and 942, respectively, but the titer enhancement was not statistically significant when PR8HA‐VLP was adjuvanted with poly(I:C) compared to the unadjuvanted PR8HA‐VLP‐immunized group (Figure 2b, right). In contrast, neutralizing titers were barely detectable 3 weeks after a single immunization of ferrets with two other recombinant HAs (A/Netherlands/399/1993 and A/Netherlands/312/2003),^21^ consistent with the enhancement of immunogenicity provided by the display of antigens from VLPs.

Since the ferret model is the gold standard to examine human influenza virus replication, the immunized ferrets were next intranasally challenged with 10^6^ plaque‐forming units (pfu) of PR8 virus to compare the duration of virus shedding. Nasal swabs were taken daily for 7 days and analyzed for infectious virus titers in plaque assays. The VLP control‐immunized group shed virus for 5 days; in contrast, the virus was eliminated by Day 3 post‐challenge in all the adjuvanted PR8HA‐VLP‐vaccinated groups (Figure 2c). Body temperature increase as a clinical symptom tended to be reduced in all the vaccinated groups, with or without adjuvants, compared to the VLP control‐vaccinated group, although there were no statistically significant differences (Figure 2d).

**FIGURE 3:**
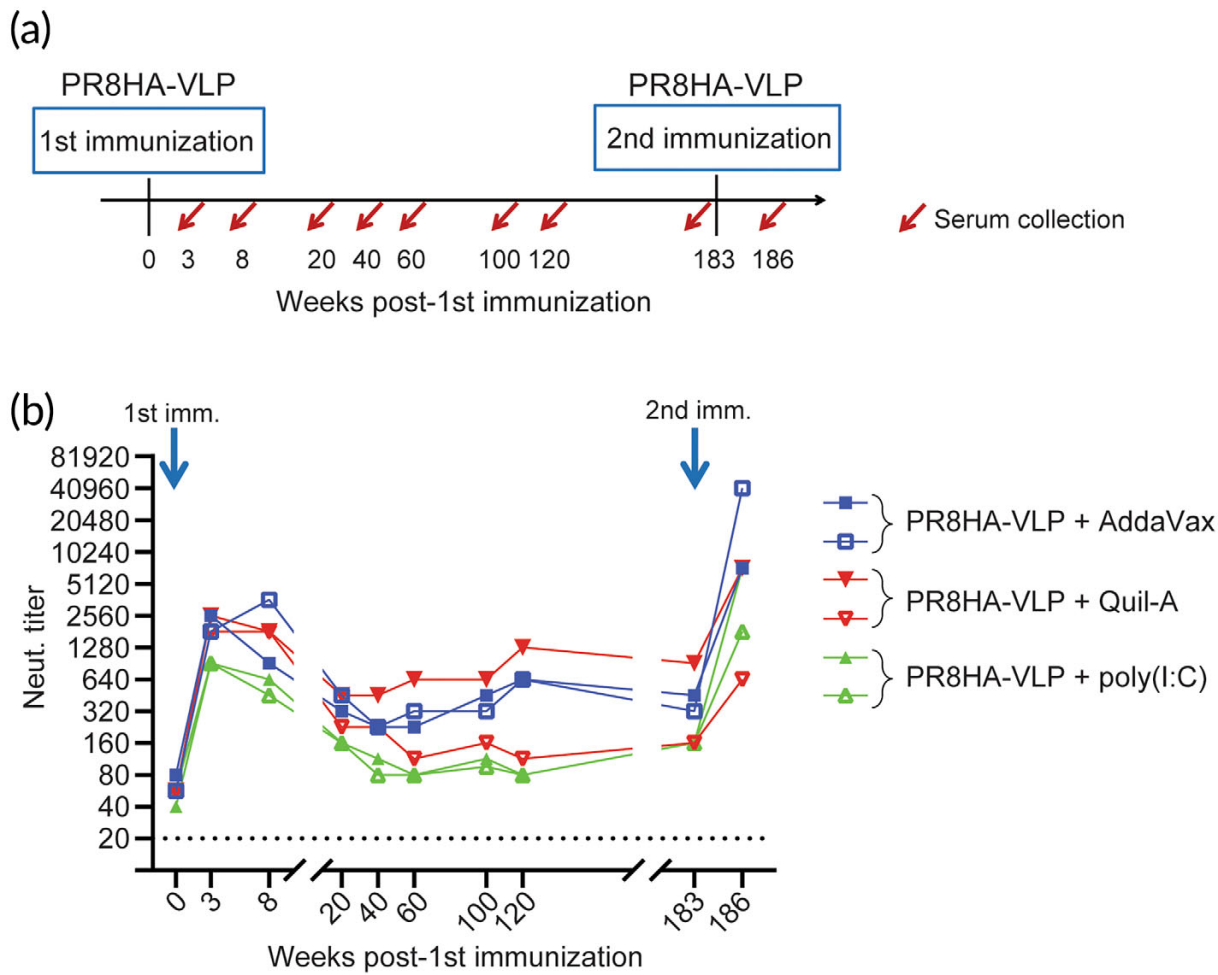
Duration of neutralizing antibody titers after single PR8HA‐VLP immunization. Ferrets were subcutaneously vaccinated with PR8HA‐VLP (HA amount: 45 μg) adjuvanted with AddaVax (500 μL), Quil‐A (30 μg), or poly(I:C) (3 μg). Serum‐neutralizing antibody titers were then monitored. At 183‐week post‐immunization, the individual animals were boosted with the identical vaccination regimen, and the neutralization titer at 3‐week post‐boost (i.e., 186 weeks after the first immunization) was analyzed. Data points show the values of individual animals connected by lines for each time point. Dashed lines represent the detection limit of the assay. VLP, virus‐like particle.

Next, we examined the persistence of the serum‐neutralizing antibodies after a single immunization with PR8HA‐VLP (Figure 3a). Ferrets were subcutaneously inoculated with PR8HA‐VLP adjuvanted with AddaVax, Quil‐A, or poly(I:C), and the serum‐neutralizing titers against the homologous PR8 virus were assessed at 3‐, 8‐, 20‐, 40‐, 60‐, 100‐, 120‐, and 183‐week post‐immunization. Although the neutralizing titers declined by 2.8‐ to 8‐fold at 20‐week post‐immunization compared to those at 8‐week post‐immunization, moderate titer levels were maintained for >3 years, especially when PR8HA‐VLP was adjuvanted with AddaVax or Quil‐A, whereas poly(I:C)‐adjuvanted immunization led to relatively lower neutralizing titers at each time point (Figure 3b).

Finally, the animals were boost‐immunized with the same regimen used for the first immunization at 183 weeks after the first immunization. At 3 weeks after the booster immunization, five of the six animals showed higher neutralizing antibody titers than those at 3 weeks after the initial immunization, suggesting that adjuvanted PR8HA‐VLP immunization is also effective for boost immunization (Figure 3b), although the small sample size is the major limitation of this study.

## 3 CONCLUSIONS

Given the enhanced immunogenicity provided by antigen display on VLPs, several previous studies have characterized the efficacy of VLPs presenting HA. Hossain et al.^22^ developed M1‐induced VLPs containing HA from the 2009 pandemic H1N1 influenza virus and showed that ferrets immunized with two doses by either intranasal or intramuscular delivery were protected from a challenge with a homologous virus. Moreover, a single dose of the VLP‐based vaccine elicited higher antibody levels than two doses of a commercial split vaccine. Sharma et al.^23^ conjugated multiple copies of the head domain of PR8 HA to VLPs from the bacteriophage P22. Immunization of mice with three doses of the VLP‐based vaccine elicited higher levels of anti‐HA antibodies than those elicited by immunization with the soluble HA head. The P22 VLP‐based vaccine also provided protection from a challenge with homologous virus. To improve the breadth of protection, Cohen et al.^24^ designed mosaic VLPs that displayed HA from various Groups 1 and 2 strains and immunized mice with two or three doses of this vaccine. The mosaic HA VLPs elicited high IgG titers against the displayed HA antigens. We recently reported a complementary strategy to increase the breadth of the anti‐HA response by inverting the orientation of HA displayed on VLPs.^10^ VLPs presenting PR8HA in an inverted orientation elicited significantly higher anti‐HA‐stalk titers than VLPs presenting PR8HA in a regular orientation. VLPs presenting PR8HA in an inverted orientation also elicited a more broadly cross‐reactive anti‐stalk response and provided enhanced protection against a challenge with a virus presenting a chimeric HA composed of a PR8HA stalk and an H6 HA head.

The longevity of the antibody response is another important component of vaccine efficacy. We therefore generated a VLP‐based influenza HA vaccine antigen and examined its immunogenicity and protective effects in ferrets. A single immunization with PR8HA‐VLP induced significantly high neutralizing antibody titers against the homologous virus with or without adjuvant. Upon virus challenge, animals vaccinated with PR8HA‐VLP in combination with AddaVax, Quil‐A, or poly(I:C) experienced a shortened infectious virus shedding period by 3 days compared to the negative control vaccinated group. Long‐term monitoring of serum‐neutralizing titers in adjuvanted vaccinated animals revealed that similar levels of neutralizing titers were retained for >3 years from 20‐week post‐immunization. Our findings further support that adjuvanted VLP‐based vaccines can be potent inducers of durable humoral immunity.

## 4 MATERIALS AND METHODS

### 4.1 Preparation of PR8HA‐VLP

PR8HA, containing a C‐terminal trimerization domain, AviTag, and hexahistidine tag, was expressed in High Five Cells, biotinylated, and purified as described previously.^7^, ^25^ MS2‐SA VLPs were assembled as previously described.^7^, ^8^, ^9^, ^10^, ^19^ SA plasmid pET21‐Streptavidin‐Glutamate_Tag (Addgene plasmid #46367) was a gift from Mark Howarth.^26^, ^27^ The optimal ratio of PR8HA to MS2‐SA was determined by analytical SEC.^10^ Various ratios were mixed together and run on SEC, and the peak corresponding to excess PR8HA was monitored.^25^ The ratio that gave the highest density of PR8HA on the VLPs without showing any excess HA was chosen. Larger volume samples were mixed at this ratio for characterization and immunization.^25^

### 4.2 Characterization of PR8HA and PR8HA‐VLP

Characterization by SDS‐PAGE and ELISA was carried out as described previously.^10^, ^11^, ^12^, ^13^ For characterization by DLS, samples were prepared by diluting 5 μg of HA, MS2‐SA, or HA on MS2‐SA to a total volume of 80 μL. Cuvettes were loaded into a Zetasizer Nano ZS (Malvern) and allowed to equilibrate to 25°C. Five measurements were taken for each sample, and the average was displayed as % Volume.

### 4.3 Cells

Madin‐Darby Canine Kidney (MDCK) cells were maintained in Eagle's minimal essential medium (MEM) containing 5% newborn calf serum.^21^ Human embryonic kidney 293T cells were cultured in Dulbecco's modified Eagle's medium (DMEM) containing 10% fetal bovine serum (FBS).^21^ SIAT1‐MDCK cells were propagated in the presence of 1 mg/mL geneticin (G418; Invivogen) in DMEM containing 5% FBS and antibiotics.^21^ These cell lines were cultured at 37°C with 5% CO_2_. All cell lines were regularly tested for mycoplasma contamination by using PCR and were confirmed to be mycoplasma‐free.^28^

### 4.4 Virus

A/Puerto Rico/8/1934 (PR8; H1N1) virus was generated by reverse genetics^29^ and propagated in MDCK cells.

### 4.5 Micro‐neutralization assay

Micro‐neutralization assays were conducted as previously described.^21^ Virus neutralization titers were determined as the reciprocal of the highest serum dilution that completely prevented cytopathic effects.^21^ Each sample was analyzed in duplicate to determine the geometric mean titers.

### 4.6 Animal experiments

All experiments with ferrets were performed in accordance with the guidelines set by the Institutional Animal Care and Use Committee at the University of Wisconsin‐Madison. The protocol was approved by the Animal Care and Use Committee of the University of Wisconsin‐Madison (protocol numbers V00806 and V6426).

### 4.7 Immunization and virus challenge of ferrets

Ferrets (4‐ to 6‐month‐old females; Triple F farm) were confirmed to be seronegative in hemagglutination inhibition assays with turkey red blood cells (seasonal H1N1, pandemic H1N1, and Flu B Victoria lineage) or neutralization assays (H3N2) with recently circulating influenza viruses before being immunized.^21^ Animals were subcutaneously immunized with 45 μg of VLP‐HA (diluted in 500 μL of phosphate‐buffered saline) adjuvanted with or without AddaVax (500 μL; InvivoGen), Quil‐A (30 μg; InvivoGen) or poly(I:C) (3 μg; InvivoGen), or immunized with VLP‐only as a negative control. The antigen was injected in the neck–shoulder, split into two to three sites to reduce the injection volume/site.^21^ Animals were bled via the jugular vein under sedation with ketamine and dexdomitor.^21^ Then, the animals were intranasally inoculated with 10^6^ pfu of PR8 virus under anesthesia.^21^ Nasal swabs were taken daily under anesthesia, alternately from the left and right nostrils, for 7 days.^28^ During this period, the body temperature was measured by using an implantable subcutaneous temperature transponder (Bio Medic Data Systems).^28^ Nasal swabs were resuspended in 1 mL of MEM containing antibiotics and kept at −80°C until titration by plaque assays in MDCK cells.^28^

## AUTHOR CONTRIBUTIONS

**Shiho Chiba:** Investigation; writing – original draft; conceptualization; formal analysis; data curation; writing – review and editing. **Tadashi Maemura:** Data curation; writing – review and editing. **Kathryn Loeffler:** Investigation; writing – original draft; formal analysis; data curation; writing – review and editing. **Steven J. Frey:** Investigation. **Chunyang Gu:** Data curation; writing – review and editing. **Asim Biswas:** Data curation; writing – review and editing. **Masato Hatta:** Data curation; writing – review and editing. **Yoshihiro Kawaoka:** Conceptualization; funding acquisition; writing – review and editing; supervision; investigation; writing – original draft. **Ravi S. Kane:** Conceptualization; funding acquisition; writing – original draft; supervision; writing – review and editing.

## CONFLICT OF INTEREST STATEMENT

YK has received unrelated funding support from Daiichi Sankyo Pharmaceutical, Toyama Chemical, Tauns Laboratories, Inc., Shionogi & Co. LTD, Otsuka Pharmaceutical, KM Biologics, Kyoritsu Seiyaku, Shinya Corporation, and Fuji Rebio. YK is a co‐founder of FluGen. The remaining authors have no conflicts of interest to declare.

## Supporting information


**Data S1:** Supporting Information.

## Data Availability

The data that support the findings of this study are available from the corresponding authors upon reasonable request.
