# Supplementary material for: Single immunization with an influenza hemagglutinin nanoparticle‐based vaccine elicits durable protective immunity
Source: Bioeng Transl Med. 2024 Jun 3;9(5):e10689. doi: 10.1002/btm2.10689 (PMC11561850; doi:10.1002/btm2.10689)
Supplement: Supplementary file 1 — Data S1: Supporting Information. [file BTM2-9-e10689-s001.pdf]

Single immunization with an influenza HA nanoparticle-based vaccine elicits durable protective immunity

Shiho Chiba[1,5], Tadashi Maemura[1], Kathryn Loeffler[2], Steven J Frey[2], Chunyang Gu[1], Asim Biswas[1], Masato Hatta[1], Yoshihiro Kawaoka[1,3,4,5*], and Ravi S Kane[2*]

[1]Influenza Research Institute, Department of Pathobiological Sciences, School of Veterinary Medicine, University of Wisconsin-Madison, WI, USA

[2]School of Chemical and Biomolecular Engineering Georgia Institute of Technology, GA, USA.

[3]Division of Virology, Department of Microbiology and Immunology, Institute of Medical Science, University of Tokyo, Tokyo 108-8639, Japan

[4]The Research Center for Global Viral Diseases, National Center for Global Health and Medicine Research Institute, Tokyo 162-8655, Japan.

[5]Pandemic Preparedness, Infection and Advanced Research Center (UTOPIA), the University of Tokyo, Tokyo 162-8655, Japan

*Correspondence:

Dr. Yoshihiro Kawaoka,

yoshihiro.kawaoka@wisc.edu

Tel: 608-265-4925

Dr. Ravi S Kane

ravi.kane@chbe.gatech.edu

Tel: 404-385-4608

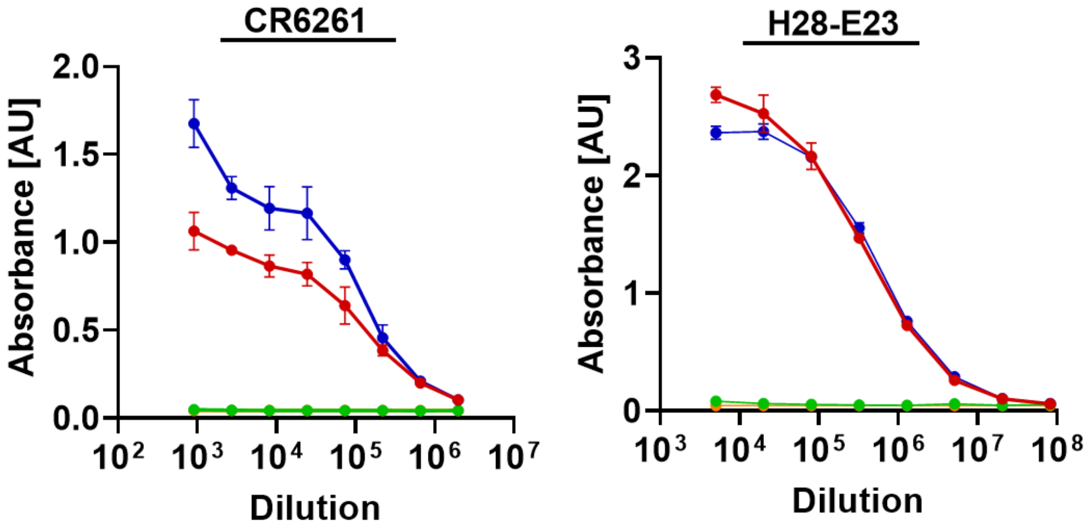

Supplementary Fig. 1. Binding curves for stalk-binding antibody CR6261 and head-binding antibody H28-E23 (Sb epitope) against PR8HA (red), PR8HA-VLP (blue), VLP control (green), and BSA (orange).

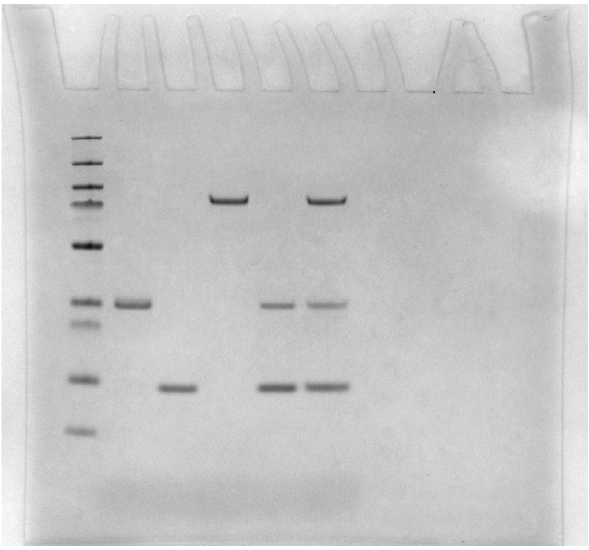

Supplementary Fig. 2. Unprocessed SDS-PAGE gel image, cropped version of which appears in Fig. 1b.
